# Ancient phylogenetic divergence of the enigmatic African rodent *Zenkerella* and the origin of anomalurid gliding

**DOI:** 10.7717/peerj.2320

**Published:** 2016-08-16

**Authors:** Steven Heritage, David Fernández, Hesham M. Sallam, Drew T. Cronin, José Manuel Esara Echube, Erik R. Seiffert

**Affiliations:** 1 Interdepartmental Doctoral Program in Anthropological Sciences, Stony Brook University, Stony Brook, NY, United States; 2 Department of Biological, Biomedical and Analytical Sciences, University of the West of England, Bristol, United Kingdom; 3 Bioko Biodiversity Protection Program, Malabo, Bioko Norte, Equatorial Guinea; 4 Mansoura University Vertebrate Paleontology Center, Department of Geology, Faculty of Science, Mansoura University, Mansoura, Egypt; 5 Department of Evolutionary Anthropology, Duke University, Durham, NC, United States; 6 Department of Biology, Drexel University, Philadelphia, PA, United States; 7 School of Environmental Sciences, National University of Equatorial Guinea, Malabo, Bioko Norte, Equatorial Guinea; 8 Department of Cell and Neurobiology, Keck School of Medicine, University of Southern California, Los Angeles, CA, United States

**Keywords:** Anomaluridae, Mammals, Phylogeny, Bioko Island, Equatorial Guinea, Patagium, Gliding, Eocene, Oligocene

## Abstract

The “scaly-tailed squirrels” of the rodent family Anomaluridae have a long evolutionary history in Africa, and are now represented by two gliding genera (*Anomalurus* and *Idiurus*) and a rare and obscure genus (*Zenkerella*) that has never been observed alive by mammalogists. *Zenkerella* shows no anatomical adaptations for gliding, but has traditionally been grouped with the glider *Idiurus* on the basis of craniodental similarities, implying that either the *Zenkerella* lineage lost its gliding adaptations, or that *Anomalurus* and *Idiurus* evolved theirs independently. Here we present the first nuclear and mitochondrial DNA sequences of *Zenkerella*, based on recently recovered whole-body specimens from Bioko Island (Equatorial Guinea), which show unambiguously that *Zenkerella* is the sister taxon of *Anomalurus* and *Idiurus*. These data indicate that gliding likely evolved only once within Anomaluridae, and that there were no subsequent evolutionary reversals. We combine this new molecular evidence with morphological data from living and extinct anomaluromorph rodents and estimate that the lineage leading to *Zenkerella* has been evolving independently in Africa since the early Eocene, approximately 49 million years ago. Recently discovered fossils further attest to the antiquity of the lineage leading to *Zenkerella*, which can now be recognized as a classic example of a “living fossil,” about which we know remarkably little. The osteological markers of gliding are estimated to have evolved along the stem lineage of the *Anomalurus*–*Idiurus* clade by the early Oligocene, potentially indicating that this adaptation evolved in response to climatic perturbations at the Eocene–Oligocene boundary (∼34 million years ago).

## Introduction

Current taxonomy recognizes the rodent family Anomaluridae with seven extant species across three genera (*Anomalurus*, *Idiurus* and *Zenkerella*) (Dieterlen, 2005; Kingdon, 2013). Living anomalurids are endemic to western and central Africa and until recently were commonly referred to as “scaly-tailed squirrels.” As the name suggests, the group’s hallmark feature is a set of scales situated on the ventral surface of the proximal tail which reportedly provide support and traction when climbing trees (Kingdon, 2013; Nowak, 1999). However, despite the vernacular, anomalurids are not closely related to the true squirrels of the rodent family Sciuridae (Fabre et al., 2012). A switch to the common name “anomalures” avoids an implied anomalurid-sciurid alliance and this term has become increasingly popular in recent years. With the exception of *Zenkerella*, living anomalurids are equipped with patagial membranes that allow them to move through forests by gliding. This adaptation evolved independently from the two other extant lineages of gliding placental mammals (i.e., dermopterans and pteromyin sciurid rodents) (Jackson & Thorington, 2012). The evolutionary pattern by which *Zenkerella* came to be differentially adapted from other anomalurids is poorly understood. In fact, *Zenkerella* is among the least studied of all mammals; virtually nothing is known of its behavior, ecology or life-history, and scarce distribution reports make it difficult to assess its conservation status. Although first described in 1898, only eleven *Zenkerella* specimens are curated in world collections (Perez del Val, Juste & Castroviejo, 1995) and to our knowledge, *Zenkerella* has never been observed alive by trained mammalogists.

Molecular phylogenetic studies recover Anomaluridae as the sister clade of the monotypic family Pedetidae (*Pedetes*, springhares) and together these families comprise the rodent suborder Anomaluromorpha (Blanga-Kanfi et al., 2009; Churakov et al., 2010; Fabre et al., 2012; Meredith et al., 2011). Like anomalurids, the two living species of *Pedetes* are African endemics. However, by contrast, springhares are adapted as burrowing terrestrial hoppers and are anatomically quite different from anomalures. Specialized details of middle ear anatomy (Meng, 1990; Ruf, Frahnert & Maier, 2009) and cranial arterial patterns (Bugge, 1974; Bugge, 1985; George, 1981) are among the few shared morphological features that support an anomalurid-pedetid clade—whereas the microstructure of incisor tooth enamel, a character classically used to affiliate rodent groups, is fundamentally different (Martin, 1993). The disparate morphotypes and molecular distances separating these families, taken in combination with the Eocene fossil record of anomalurid evolution in Africa (Marivaux et al., 2015; Sallam, Seiffert & Simons, 2010; Sallam et al., 2010), seemingly indicate an ancient divergence of anomaluromorph lineages on that continent.

Extant anomalurids are arranged in two subfamilies. Anomalurinae includes four species placed in the genus *Anomalurus*—although craniodental distinctions have prompted some authors to advocate the transfer of *Anomalurus beecrofti* into a different genus, *Anomalurops* (Kingdon, 2013). Both species of *Idiurus* and the monotypic *Zenkerella* (*Zenkerella insignis*) are grouped in the subfamily Zenkerellinae (Dieterlen, 2005). Previous authors have applied the synonym Idiurinae to ally these two genera (Dieterlen, 2005; Nowak, 1999). Characters common to zenkerelline taxa include an origin of the lower maxillary process close to the incisors (cf. closer to the cheek teeth in *Anomalurus*), a relatively deep dorsoventral dimension of the premaxilla (cf. relatively shallow in *Anomalurus*) and a notch on the lingual surface of the upper incisors (cf. unnotched in *Anomalurus*) (Kingdon, 2013).

If this taxonomic arrangement reflects phylogenetic descent, there are implications for how to interpret the evolution of patagial gliding. *Anomalurus* and *Idiurus* are unique among mammalian gliders in having an attachment of the patagium to a cartilaginous support rod that extends from the elbow (Coster et al., 2015). By contrast, pteromyin sciurids have a support cartilage that extends from the wrist (Jackson & Schouten, 2012). In gliding anomalurids, the structure is approximately the length of the forearm and projects anterolaterally from a bony expansion of the ulnar olecranon process. *Zenkerella* lacks this osteological feature and the patagia and cartilaginous struts are absent. At least three evolutionary scenarios are plausible (Coster et al., 2015). Gliding could have evolved prior to the common ancestor of living anomalurids and was subsequently lost in the *Zenkerella* lineage. Alternatively, from a non-gliding ancestor, *Anomalurus* and *Idiurus* could be convergently adapted while *Zenkerella* has retained the ancestral condition. A third scenario is that *Anomalurus* and *Idiurus* are more closely related to each other than either is to *Zenkerella*, in which case patagial gliding is more likely to have evolved once, along the stem lineage of the *Anomalurus*–*Idiurus* clade. This third scenario, however, is incongruent with the current classification and phylogenetic hypotheses. A robust phylogenetic framework is required to evaluate these competing hypotheses, but the paucity and preservation of available specimens have been limiting factors for incorporating *Zenkerella* into molecular phylogenetic analyses.

As part of an ongoing biodiversity monitoring and conservation program on Bioko Island, Equatorial Guinea, we have recently recovered three additional *Z. insignis* specimens. With newly generated nuclear and mitochondrial sequences from two of these individuals, here we test the proposed phylogenetic relationships of *Zenkerella* using a Bayesian approach. After combining the molecular data with morphological data from living and fossil taxa, we then use Bayesian “tip-dating” methods to estimate divergence dates within Anomaluromorpha. With these results, we reassess the taxonomic placement of *Zenkerella* and suggest a scenario for the evolution of gliding in anomaluromorph rodents.

## Methods

### Specimens

The three new *Z. insignis* specimens are whole animals deposited at the Duke Lemur Center Division of Fossil Primates at Duke University (Durham, NC, USA). Specimen voucher numbers are DPC 91000–91002. The animals were caught in ground snares by local trappers in the proximity of Ureca village near the southern tip of Bioko Island, Equatorial Guinea. Specimens were collected and exported under permits issued to the Bioko Biodiversity Protection Program (BBPP) (field permit 154/2015 from the Equatorial Guinea Department of Culture and Tourism; export permit 169/2015 from the National University of Equatorial Guinea). DPC 91000 (male) was collected July 2014 at GPS coordinates ∼N3.25469 E8.58544; DPC 91001 (male) and DPC 91002 (female) were collected in September 2015 and October 2015 at ∼N3.24999 E8.56825 and ∼N3.25627 E8.58408, but trappers did not note sex upon recovery and were unable to associate these individuals to specific sites. While in Bioko, the earlier specimen was preserved in formalin for 11 weeks and then transferred to 70% ethanol. The more recently recovered specimens were preserved in 70% ethanol upon collection and later transferred to a molecular biology grade 70% ethanol which is certified DNase, RNase and protease free.

### DNA extraction and purification

Total DNA was extracted from DPC 91001 and DPC 91002 using a Qiagen DNEasy Blood and Tissue Kit and protocol. Cheek swabs were used to collect buccal epithelial cells as starting genetic material. Specimens were handled using sterile gloves and instruments. For the lysis step, we elected to use 40 µl of Proteinase K, 360 µl of buffer ATL and to incubate at 56 °C for 24 h. For the purification steps, we followed kit protocol and eluted the spin column membrane twice to increase total yield. A negative control was processed in parallel following identical protocol but starting with a sterile swab.

### Amplification and sequencing

Two nuclear (*IRBP, VWF*) and three mitochondrial (*12S, COX1, CYTB*) loci were targeted for PCR amplification. Oligonucleotide primers were newly designed using a comparative sample of existing anomaluromorph and other rodent sequences retrieved from GenBank. Amplifications were performed using Promega GoTaq G2 Hot Start Master Mix and protocol for 25 µl reactions. PCR was run for 40 cycles with each cycle consisting of denaturation (1 min, 95 °C), annealing (1 min, variable temperature) and extension (variable time, 72 °C). The first cycle was preceded by an initial denaturation (5 min, 95 °C) and the last cycle was followed by a final extension (5 min, 72 °C). Primer sequences, annealing temperatures and extension times are reported in Data S1. PCR products were electrophoresed on a 1% agarose gel to screen for expected lengths and non-specifics. All negative control lanes were blank. DNA bands in excised gel slices were purified and sequenced twice at Eurofins MWG Operon (Louisville, KY, USA) using an Applied Biosystems 3730xl DNA Analyzer. To screen for possible contamination, *Zenkerella* sequence results were compared to available orthologs from *Anomalurus*, *Idiurus*, *Pedetes* and all mammalian taxa that have been previously processed in the lab. In all cases, *Zenkerella* sequences were unique and shared a higher percent identity with other anomaluromorphs than with non-anomaluromorph sequences. *Zenkerella* sequences are deposited at GenBank under accession numbers KU900240–KU900249.

### DNA dataset

Our DNA dataset comprises five gene segments across 67 rodent and 11 outgroup taxa. All rodent suborders and families are represented. To improve data completeness, 14 non-anomaluromorph operational taxa combine congeneric sequences. Accession numbers for sequences retrieved from GenBank and identifications of chimeric operational taxa are in Data S2. Alignments were performed using the Geneious (v7.1.7) (Kearse et al., 2012) multiple alignment tool with default settings. For the four amino acid coding sequences, some positions in the alignments were manually adjusted using the translation frame as a guide. Translated sequences contained no stop codons. For the rRNA sequence, regions of alignment ambiguity were manually removed. Individual gene alignments were concatenated using Geneious yielding a 5,906 base pair alignment which is available in Data S3.

### Phylogenetic analysis

PartitionFinder (v1.1.1) (Lanfear et al., 2012) was used to select a subset scheme and substitution models as assessed by the Bayesian Information Criterion (BIC). Input data blocks were defined by gene and codon positions. Settings specified exploration of all models available in MrBayes and all subset schemes given the data blocks. The best-fit scheme proposed eight subsets: (*12S*, *CYTB*-pos1) (*COX1*-pos1) (*COX1*-pos2, *CYTB*-pos2) (*COX1*-pos3) (*CYTB*-pos3) (*IRBP*-pos1, *VWF*-pos1) (*IRBP*-pos2, *VWF*-pos2) (*IRBP*-pos3, *VWF*-pos3). The best-fit model for subsets 1–7 was GTR+I+G and for subset 8 was GTR+G.

MrBayes (v3.2.6) (Ronquist et al., 2012b) was used to infer phylogenetic relationships. Partitions and models were set according to PartitionFinder results and model parameters were unlinked. Two MCMCMC runs were called with four chains each (three hot, one cold) using sample frequency 1000 over 50 million generations. Heating temperature was set to 0.05 and relative burn-in to 0.25. We assessed adequate run length using three diagnostics. (1) Minimum effective sample sizes (ESS) for all parameters were >200 with a mean of 11,254. (2) The average standard deviation of split frequencies (ASDSF) was 0.000954. (3) Across all parameters, the mean potential scale reduction factor (PSRF) was 1.000487 with a maximum |1-PSRF| of 0.006835. We interpret these values as indication that parameter space was sufficiently sampled and the runs had converged.

In addition to Bayesian inference, we used RAxML (v8.2.7) (Stamatakis, 2014) to perform maximum likelihood (ML) analyses using the concatenated alignment and each individual marker. Appropriate subset and model choices were discerned with PartitionFinder. Full methodological details of ML runs are in Data S4.

### Clock analysis

To estimate divergence times, we used a total-evidence Bayesian “tip-dating” approach (Ronquist et al., 2012a; Ronquist et al., 2012b) implemented in MrBayes. With the exception of *Pedetes surdaster*, all anomaluromorph species in the molecular dataset are scored for morphological characters in the matrix of Marivaux et al. (2015). That character matrix also includes fossil data for three anomalurid and four nementchamyid / nonanomalurid taxa which bear on the chronology of the suborder. We expanded the fossil sample by using the same character descriptions and interpretations of crest homologies to newly score *Zenkerella wintoni* (Lavocat, 1973), *Prozenkerella saharaensis* (Coster et al., 2015) and *Propedetes efeldensis* (Mein & Pickford, 2008). Our version of the morphological matrix includes 15 anomaluromorph taxa and 150 (mostly dental) characters. The molecular and morphological datasets were concatenated using Mesquite (v3.04) (Maddison & Maddison, 2015) and the combined evidence matrix is available in Data S5.

A gamma distributed Markov *k* model was assigned to the morphological partition using default priors and variable coding. Following Marivaux et al. (2015), 95 morphological characters were treated as ordered. Model settings for the molecular partition were identical to those used in the non-clock analysis. For fossil “tip” calibrations, age uncertainty was accommodated by employing uniform priors based on (when possible) the currently recognized upper and lower bounds of magnetochrons in which species might reasonably be placed. Data S6 reports the chronology and provenance of included fossil taxa. Using the molecular tree branching pattern, hard topological constraints were applied to all taxa outside the anomaluromorph suborder. For anomaluromorphs, partial constraints were applied to extant species to serve as a scaffold for the placement of fossils. We selected the independent gamma rates (IGR) model to estimate relaxed clock rate variation and used a uniform distribution for the branch lengths prior.

Non-rodent taxa were excluded from the clock analysis rendering the crown rodent node and tree root as synonymous. Previous cladistic testing has positioned the fossil taxon *Paramys* within the rodent crown group (Benton et al., 2015; Marivaux, Vianey-Liaud & Jaeger, 2004). The earliest appearances of *Paramys* in the latest Paleocene signals a minimum age for the crown rodent node (Benton et al., 2015; Rose, 1981). The earliest Paleocene marks the first appearance of fossil taxa definitively positioned within the placental mammal crown group. Included are the laurasiatherian *Protungulatum*, euarchontan *Purgatorius* and glirans *Mimotona* and *Heomys* (O’Leary et al., 2013). From a paleontological perspective, we find it reasonable to assume that the relatively nested crown rodent node postdates the earliest known crown placentals. Therefore the maximum and minimum of the uniformly distributed tree age prior were set to correspond to the beginning (66 Ma) and end (56 Ma) of the Paleocene epoch. We recognize that many molecular-based estimates for the age of the crown rodent node extend into the Cretaceous. To accommodate this possibility an alternate analysis was run after lifting the K-Pg limit of the tree age prior.

The clock rate prior is an initial estimate for a distribution describing the number of substitutions per site per million years. To derive this estimate, we first used the non-clock molecular tree and the dist.nodes function from the R package APE (v3.4) (Paradis, Claude & Strimmer, 2004) to extract path lengths from each rodent tip to the rodent node. Next, each path length was divided by the center of the tree age prior. The fitdist function from the R package fitdistrplus (v1.0-6) (Delignette-Muller & Dutang, 2015) was then used to fit gamma, log-normal and normal distributions to the set of scaled path lengths. A comparison of BIC scores was used to discern the log-normal distribution as best fit. With this result, we estimated the clock rate prior mean at −2.646972 with a 0.202816 standard deviation. The heating temperature was lowered to 0.02 to promote chain swapping. With the exception of generations, all other MCMCMC settings were identical to the non-clock analysis.

To choose an IGR variance prior, we ran several pilot analyses using exponential distributions with values ranging from 5 to 35 in increments of 5. Pilots were run for 10 million generations applying the stepping-stone method. Marginal likelihoods and select node dates were compared to investigate the effect of the prior value. Tree topologies across all pilots were identical. The range of marginal likelihoods was 1.73. The range of mean age estimates for the crown rodent and crown anomaluromorph nodes were 1.21 and 0.93 million years, respectively. We interpret these findings as negligible variation and suspect that longer, more densely sampled analyses would converge. When applied to our combined evidence dataset, clock analysis seems mostly insensitive to a prior value for exponential IGR variance—at least given the other analysis settings. Therefore, we selected the MrBayes default value of 10.

The final clock analyses (rodent node with and without K-Pg maximum limit) were run for 35 million generations each. Again, adequate run length, sampling and convergence were assessed with three diagnostics. (1) Minimum ESS for all parameters were >200 with means of 8280 and 8488. (2) The ASDSFs were 0.007418 and 0.006440. (3) The parameter mean PSRFs were 1.005415 and 1.001295.

## Results

### Specimens

**Figure 1:**
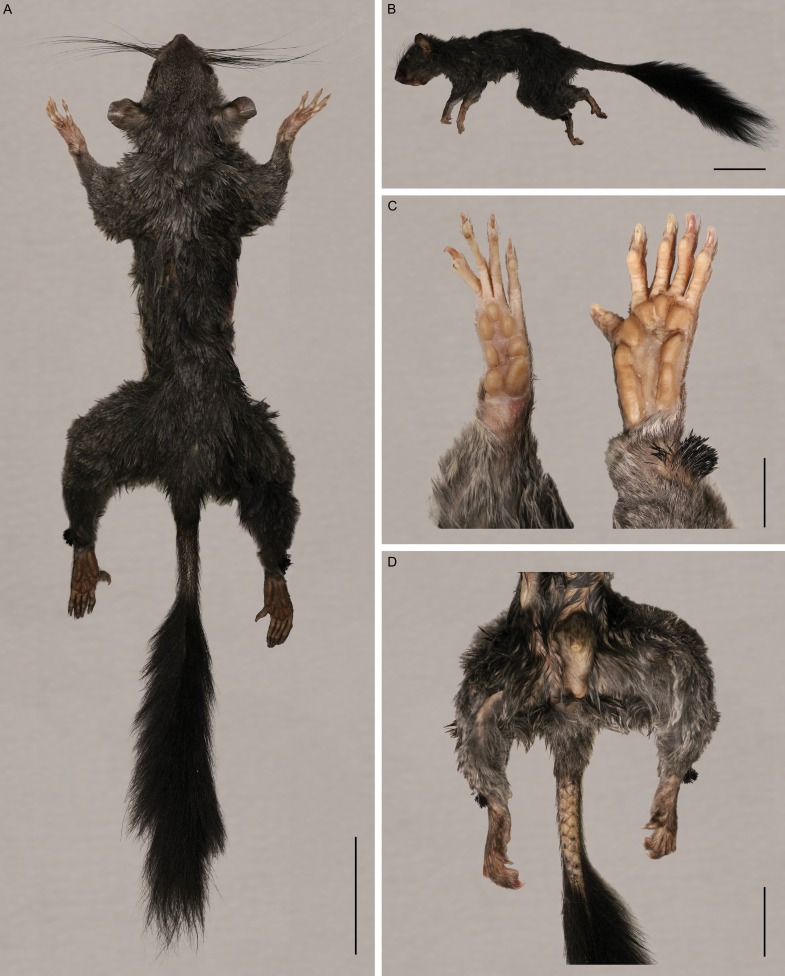
Photographs of *Zenkerella insignis* DPC 91001 male. (A) dorsal view, scale bar 50 mm. (B) lateral view, scale bar 50 mm. (C) palmar view of right hand and plantar view of left foot, scale bar 10 mm. (D) ventral view of proximal tail and genitals, scale bar 25 mm.

**Figure 2:**
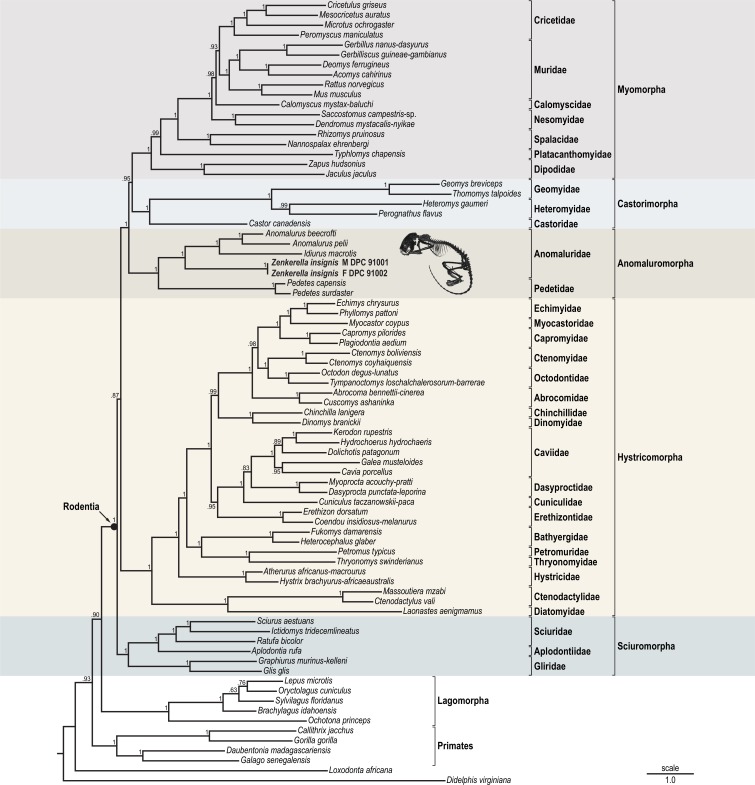
Result of Bayesian phylogenetic analysis of the 5,906 base pair concatenated DNA alignment. Two nuclear (*IRBP, VWF*) and three mitochondrial (*12S, COX1, CYTB*) sequences were included. Values at nodes are posterior probability (=clade credibility). Branch lengths are mean values from the posterior distribution representing expected number of substitutions per site. Rodent suborder and family designations follow the current taxonomy of Wilson & Reeder (2005).

We compared preliminary micro-CT images of DPC 91000 to Fig. 9 from Marivaux et al. (2011) and Figs. 2 and 6 from Coster et al. (2015) and confirm that this specimen shares the craniodental and postcranial morphological features that distinguish *Zenkerella*. Compared to other living anomalures, *Zenkerella* exhibits a highly derived molar morphology that is characterized by simple loph and lophid patterns. Unlike *Anomalurus* and *Idiurus*, the molar occlusal surfaces of *Zenkerella* are surrounded by a continuous external crest that, in occlusal view, is relatively round in shape (Coster et al., 2015). *Zenkerella* molars are distinct in exhibiting a single transverse crest that subdivides the occlusal surface into two foveae. *Zenkerella* is also distinguished from other living anomalures in the relative sizes of the upper cheek teeth. P4 and M3 are similar in size and both are smaller than M1 and M2 (Kingdon, 2013). The rows of scales on the ventral tail (Fig. 1) are diagnostic for anomalurids and the lack of patagia is unique to *Zenkerella* (Kingdon, 2013). Previous reports of *Zenkerella*’s geographic distribution include Bioko near Ureca (Perez del Val, Juste & Castroviejo, 1995). Our comparison to the craniodental and postcranial features of mainland specimens (Marivaux et al., 2011; Coster et al., 2015) finds that at present there is no compelling reason to identify the Bioko Island population as a new species.

Literature accounts of new *Z. insignis* data are especially rare, prompting our report of basic metrics, description and ecological information. For the three new specimens, head and body lengths are 181–198 mm and tail lengths are 168–184 mm. These measurements are within or slightly larger than previously reported ranges (Kingdon, 2013; Nowak, 1999). We cannot confirm that all specimens are full adults. Body hair is gunmetal (or ashy) grey and tail hair is black and bushy. Hairs of the lateral ankle tufts are black, coarse and spiky. Maximum bilateral vibrissae span is approximately six times the head width. The scaly portion of the tail extends for 20–25% of tail length. Hands and feet have four and five digits respectively and claws are present on all digits. The first toe (pedal digit I) is somewhat divergent. The scrotum is relatively large and CT images indicate that a baculum is present. The female has 4 nipples and our dissection of the abdomen confirms that she is not pregnant. Specimen photographs are in Fig. 1. Entrapment in ground snares indicates that *Zenkerella* is not exclusively arboreal. When interviewed, villagers in the vicinity of the collection site reported that *Zenkerella* is infrequently (once or twice a year) caught in forest floor traps and that the species is not desirable for meat. Interviewees also suggested that *Zenkerella* is nocturnally active and sleeps in tree hollows. Additional details of anatomy and ecology will be part of forthcoming research.

### Phylogeny

Bayesian analysis of the concatenated nuclear and mitochondrial alignment strongly supports monophyly for each of the five currently recognized rodent suborders and the pattern of basal divergence branches into three major lineages. The suborder Sciuromorpha derives from the earliest split and is positioned as sister to all other living rodents. The remaining two lineages are the suborder Hystricomorpha and its sister-clade which comprises the suborders Myomorpha, Castorimorpha and Anomaluromorpha. This basal divergence pattern among the three lineages is consistent with previous retroposon analyses and very large scale DNA sequence analyses (Churakov et al., 2010; Fabre et al., 2012).

With all anomaluromorph genera and all rodent families represented, our results recover Anomaluridae and Pedetidae as sister taxa; all anomaluromorph splits were inferred with maximum posterior probabilities (Fig. 2). Among anomalurids, the genera *Idiurus* and *Anomalurus* are sister taxa to the exclusion of *Zenkerella*. This finding is incompatible with the current classification of *Zenkerella* and *Idiurus* in the subfamily Zenkerellinae, but is consistent with the relationships proposed by Tullberg (1899). A taxonomic revision is discussed below.

ML analysis of the concatenated alignment resulted in a topology which is identical to the Bayesian tree and all anomaluromorph nodes are supported with very strong bootstrap proportions. ML analyses of all individual markers recovered monophyletic anomaluromorph groups to the exclusion of other rodents. Individual marker analyses also inferred relationships among anomaluromorphs that are fully congruent with the concatenation trees. These results detect no conflict in phylogenetic signal between nuclear and mitochondrial sequences. Data S4 includes ML results.

### Divergence time

**Figure 3:**
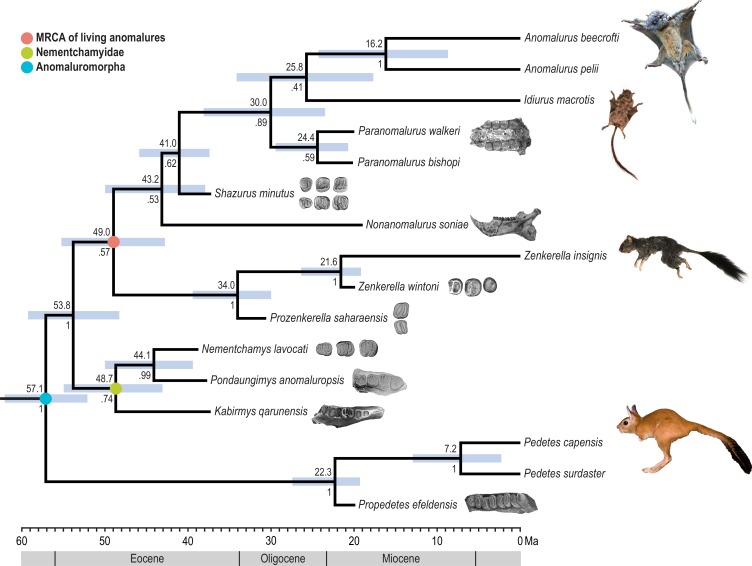
Result of Bayesian time-tree analysis using the fossil tip-dating method applied to combined molecular and morphological data. Time scale is in millions of years. Values above nodes indicate mean highest posterior density age estimates. Bars underlying nodes represent 95% age ranges which accommodate topological uncertainty. Values below nodes are posterior probabilities of splits. Images of fossil specimens are modified after Coster et al. (2015, *Prozenkerella saharaensis*), Jaeger et al. (1985, *Nementchamys lavocati*), Lavocat (1973; *Zenkerella wintoni*), Marivaux et al. (2005, *Pondaungimys anomaluropsis*), Mein & Pickford (2008; *Propedetes efeldensis*), Pickford et al. (2013; *Nonanomalurus soniae*), Sallam, Seiffert & Simons (2010; *Shazurus minutus*), and Sallam et al. (2010; *Kabirmys qarunensis*).

Tip-dating analysis using molecular and morphological data from living and fossil taxa estimates an Eocene date for the most recent common ancestor of extant anomalures (Fig. 3). The mean highest posterior density of this estimate is at 49.0 Ma with a 95% range (55.2 to 42.8 Ma) through the early and early middle Eocene. The node representing the most recent common ancestor of *Anomalurus* and *Idiurus* has a mean estimate at 25.6 Ma with a 95% range (34.2 to 17.7 Ma) spanning the Oligocene and into the early Miocene.

For fossil taxa, the topological estimate of our combined-evidence clock analysis is identical or nearly identical to recent parsimony analyses that sample similar taxa and morphological characters (Coster et al., 2015; Marivaux et al., 2011; Marivaux et al., 2015), but the phylogenetic position of *Zenkerella* (and its putative close relative *Prozenkerella*) is novel. A comparison is in Data S7. The previous parsimony applications have yielded relatively low clade support for splits among fossil species. While our topology for extant anomaluromorphs is supported by 100% clade credibility at all nodes, posterior probabilities for fossil relationships are suboptimal. However, a major advantage of the Bayesian method is that topological uncertainty is accommodated in age-range estimates. Given the constraints of the tree age prior, estimated divergence time ranges for living species should be robust despite alternate possibilities for relationships among fossils.

We included only undisputed anomaluromorph fossil taxa in the clock analysis and therefore all fossil calibrations were within the suborder. A K-Pg constraint for the maximum age of the crown rodent node served as an external calibration. Results of an alternate analysis which lifted this limit resulted in Cretaceous estimates for crown rodents, crown anomaluromorphs and crown anomalurids. From a paleontological perspective we consider these dates to be unrealistic; the anomaluromorph presence in Africa is almost certainly due to an early Paleogene dispersal across the Tethys Sea, and there are no rodents in the African fossil record before the early-middle Eocene boundary (Marivaux et al., 2015). Methodologically, the absence of an informed constraint for the maximum age of crown rodents may be countered by the inclusion of multiple fossil taxa from other rodent suborders and mammalian orders. As this falls beyond the scope of the current study, we caution that the alternate time-tree modeled without the external reference may be unreliable.

## Discussion

These new *Z. insignis* individuals are the first field reports of the species in over 20 years. Importantly, we can confirm *Zenkerella* distribution near a BBPP research site, and efforts have been initiated to investigate *Zenkerella*’s mostly unknown basic biology. The results presented here provide necessary phylogenetic context. The specimen count in world collections is now fourteen. As whole animals originally preserved in ethanol, these may be the only *Zenkerella* museum specimens that can provide long molecular sequence reads. However, methods to extract archival DNA from older dried and formalin-fixed tissues are improving. Accordingly, we have begun amplifying additional nuclear and mitochondrial targets to be made available in future studies. Furthermore, we are optimistic that additional specimens will be recovered in the future as local trappers have been made aware of their importance for scientific research.

We recommend a taxonomic revision of the family Anomaluridae. The genera *Zenkerella* and *Idiurus* were originally grouped in the subfamily Zenkerellinae without explicit phylogenetic testing. This arrangement is inconsistent with our phylogenetic results which recover strong support for an *Anomalurus*–*Idiurus* clade that is sister to *Zenkerella*. We therefore advocate removal of *Idiurus* from the Zenkerellinae.

*Idiurus* has previously warranted subfamilial distinction from the Anomalurinae (Matschie, 1898). Our mean estimate for the *Anomalurus*–*Idiurus* divergence is late Oligocene. Independent analyses of mammalian phylogenetic chronology have estimated several rodent sister families as diverging around the Oligocene–Miocene transition and some more recently (e.g., Ctenomyidae–Octodontidae, Echimyidae–Myocastoridae, Petromuridae–Thryonomyidae, Muridae–Cricetidae) (Meredith et al., 2011). Therefore, placing *Idiurus* in the subfamily Anomalurinae is not justifiable. We suggest resurrecting Idiurinae (Miller & Gidley, 1918) as a valid subfamily to include the genus *Idiurus*.

With an estimated phylogenetic divergence deep in the Eocene, we argue that *Zenkerella* should be placed in its own family, Zenkerellidae, and that all of the anomalures should be encompassed within a superfamily, Anomaluroidea. All fossil taxa sharing the most recent common ancestor of *Z. insignis* and *A. beecrofti* should be considered part of the crown superfamily. Our proposed taxonomic revision of extant anomalures is as follows:

**Table:** 

SUPERFAMILY Anomaluroidea Gervais, 1849
FAMILY Anomaluridae Gervais, 1849
SUBFAMILY Anomalurinae Gervais, 1849
*Anomalurus* Waterhouse, 1843
*Anomalurus beecrofti* Fraser, 1853
*Anomalurus derbianus* (Gray, 1842)
*Anomalurus pelii* (Schlegel & Müller, 1845)
*Anomalurus pusillus* Thomas, 1887
SUBFAMILY Idiurinae Miller & Gidley, 1918 new rank
*Idiurus* Matschie, 1894
*Idiurus macrotis* Miller, 1898
*Idiurus zenkeri* Matschie, 1894
FAMILY Zenkerellidae Matschie, 1898 new rank
*Zenkerella* Matschie, 1898
*Zenkerella insignis* Matschie, 1898

The phylogenetic position of *Zenkerella* as the sister taxon of other living anomalures and the ancient divergence of the lineage leading to *Zenkerella*, combined with the evidence provided by recently discovered anomaluroid fossils, helps to clarify several aspects of the group’s deep evolutionary history in Africa. First, our results are consistent with a single origin of patagial gliding along the shared stem lineage of *Anomalurus* and *Idiurus*. Early Miocene *Paranomalurus* from fossil sites in Uganda and Kenya already show the characteristic expanded ulnar olecranon process that correlates with the presence of a styliform cartilaginous support for the patagial gliding membrane (Pickford et al., 2013), and our tip-dating analysis estimates that this gliding *Anomalurus–Idiurus–Paranomalurus* clade originated in the early Oligocene (∼30 Ma). These results open up the possibility that anomalurid gliding adaptations evolved during a particularly turbulent “icehouse” phase in the Earth’s climatic and biotic history that was marked by the rapid onset of global cooling and, in northern Africa, the local extinction of thermophilic strepsirrhine primates (Seiffert, 2007). Fossil members of the *Anomalurus–Idiurus–Paranomalurus* clade have never been recovered from the well-sampled early Oligocene deposits of the Fayum Depression of Egypt, potentially indicating that the range of this radiation of gliding anomalurids was restricted to more equatorial parts of Africa.

Just as remarkable, our estimate of an early or middle Eocene divergence of crown anomaluroids indicates that the *Zenkerella* lineage has been independently evolving in Africa for almost 50 million years. The recent discovery of isolated teeth of a ∼31 Ma zenkerellid, *Prozenkerella saharaensis* (Coster et al., 2015), in Libya provides further support for this scenario, and also reveals that zenkerellids were able to persist through early Oligocene paleoenvironments where gliding anomalurids have never been found. The tooth morphology of *P. saharaensis* also indicates that zenkerellids’ simple molar structure evolved very early in that clade’s evolutionary history and remained highly conserved for tens of millions of years. The presence of the genus *Zenkerella* in ∼20 Ma deposits in east Africa (Lavocat, 1973) further indicates that *Zenkerella* is a true “living fossil” about which we know remarkably little. Analyses in preparation on the skeletal and muscular morphology of *Zenkerella* will allow us to generate hypotheses about the locomotion and positional behavior of *Z. insignis* that can be tested through future field studies on Bioko Island.

The early-middle Eocene estimate for the origin of crown Anomaluroidea also indicates that the presumably homologous shared behavioral and anatomical features of extant anomaluroids likely trace back to this ancient phase in Africa’s biotic history. The scales on the ventral surface of anomaluroids’ tails, which reportedly provide traction in arboreal settings, and reports from Bioko trappers that *Zenkerella* might be nocturnal and sleep in tree hollows like *Anomalurus* and *Idiurus*, suggest that the ancestral anomaluroid would have also been a nocturnal, and at least partially arboreal, mammal that utilized trees as sleeping sites during the day. An arboreal and nocturnal lifestyle in early anomaluroids might have allowed members of this clade to avoid direct competition with the presumably largely terrestrial, and possibly largely diurnal, hystricognathous rodents that dispersed into Afro-Arabia in the middle Eocene, and underwent a very successful adaptive radiation during the later Paleogene (Marivaux et al., 2014; Sallam & Seiffert, 2016; Sallam et al., 2009). Anomaluroids are the only mammals aside from anthropoid and strepsirrhine primates (and perhaps some bat lineages) that can, by comparison with living members, be identified as probable occupants of arboreal niches in the Paleogene of Africa.

##  Supplemental Information

10.7717/peerj.2320/supp-1 Data S1 Primer sequences, annealing temperatures and extension times

10.7717/peerj.2320/supp-2 Data S2 Accession numbers for DNA sequences retrieved from GenBank

10.7717/peerj.2320/supp-3 Data S3 Molecular data matrix, MrBayes settings, and non-clock tree

10.7717/peerj.2320/supp-4 Data S4 Maximum likelihood analyses of molecular data

10.7717/peerj.2320/supp-5 Data S5 Combined data matrix for tip-dating analysis, MrBayes settings, and clock tree

10.7717/peerj.2320/supp-6 Data S6 Chronology and provenance of fossils used in tip-dating analysis

10.7717/peerj.2320/supp-7 Data S7 Comparison of trees from morphology-based analyses

## Additional Information and Declarations

### Field Study Permissions

The following information was supplied relating to field study approvals (i.e., approving body and any reference numbers): Equatorial Guinea Department of Culture and Tourism—154/2015 (Field Permit) UNGE—Dr. Maximiliano Fero Meñe—169/2015 (Export) Department of Culture and Tourism—Guillermina Mekuy Mba Obono (Field Permit)

### DNA Deposition

The following information was supplied regarding the deposition of DNA sequences: GenBank accession numbers:
KU900240, KU900242, KU900244, KU900246, KU900248, KU900241, KU900243, KU900245, KU900247, KU900249.

### Data Availability

The following information was supplied regarding data availability: The raw data has been supplied as Supplemental Files.
