# Supplementary material for: Ancient phylogenetic divergence of the enigmatic African rodent Zenkerella and the origin of anomalurid gliding
Source: PeerJ. 2016 Aug 16;4:e2320. doi: 10.7717/peerj.2320 (PMC4991859; doi:10.7717/peerj.2320)

Ancient phylogenetic divergence of the enigmatic African rodent *Zenkerella* and the origin of anomalurid gliding

Heritage S, Fernández D, Sallam HM, Cronin DT, Esara Echube JM, Seiffert ER (2016)

Data S4. Maximum likelihood analyses of molecular data.

The concatenated alignment and each individual marker were also analyzed using ML methods. PartitionFinder was used to test subset schemes and substitution models with optimal choices discerned by the BIC. Results of these tests are as follows:

(A) 12S, COX1, CYTB, IRBP, VWF - concatenation
GTR+I+G
Subset1 = 12S, CYTB_pos1
Subset2 = COX1_pos1
Subset3 = COX1_pos2, CYTB_pos2
Subset4 = COX1_pos3
Subset5 = CYTB_pos3
Subset6 = IRBP_pos1, VWF_pos1
Subset7 = IRBP_pos2, VWF_pos2
Subset8 = IRBP_pos3, VWF_pos2

(B) 12S - mitochondrial
GTR+I+G
Subset1 = 12S

(C) COX1 - mitochondrial
GTR+I+G
Subset1 = COX1_pos1
Subset2 = COX1_pos2
Subset3 = COX1_pos3

(D) CYTB - mitochondrial
GTR+I+G
Subset1 = CYTB_pos1
Subset2 = CYTB_pos2
Subset3 = CYTB_pos3

(E) IRBP - nuclear
GTR+G
Subset1 = IRBP_pos1
Subset2 = IRBP_pos2
Subset3 = IRBP_pos3

(F) VWF - nuclear
GTR+I+G
Subset1 = VWF_pos1
Subset2 = VWF_pos2
Subset3 = VWF_pos3

Phylogenetic relationships were inferred using RAxML. Partition schemes and substitution models were set according to PartitionFinder results. For each analysis, 100 runs and 1000 thorough bootstrap replicates were called. For the five analyses of individual markers, we also loaded ordinal level constraint trees. Alignments, partition schemes, constraint trees and result trees are included in Data S4. ML and Bayesian analyses of the concatenated alignment resulted in identical topologies.

(A) 12S, COX1, CYTB, IRBP, VWF - concatenation

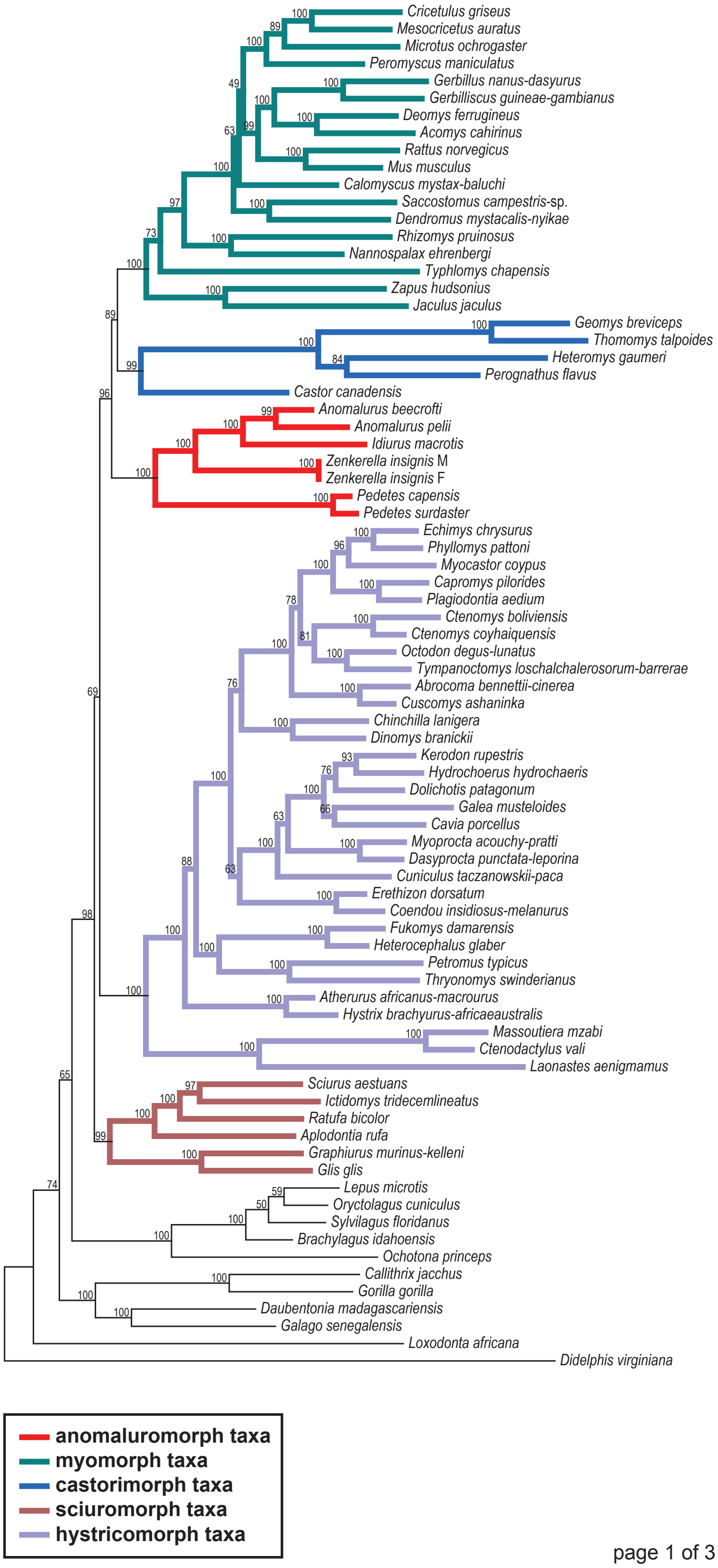

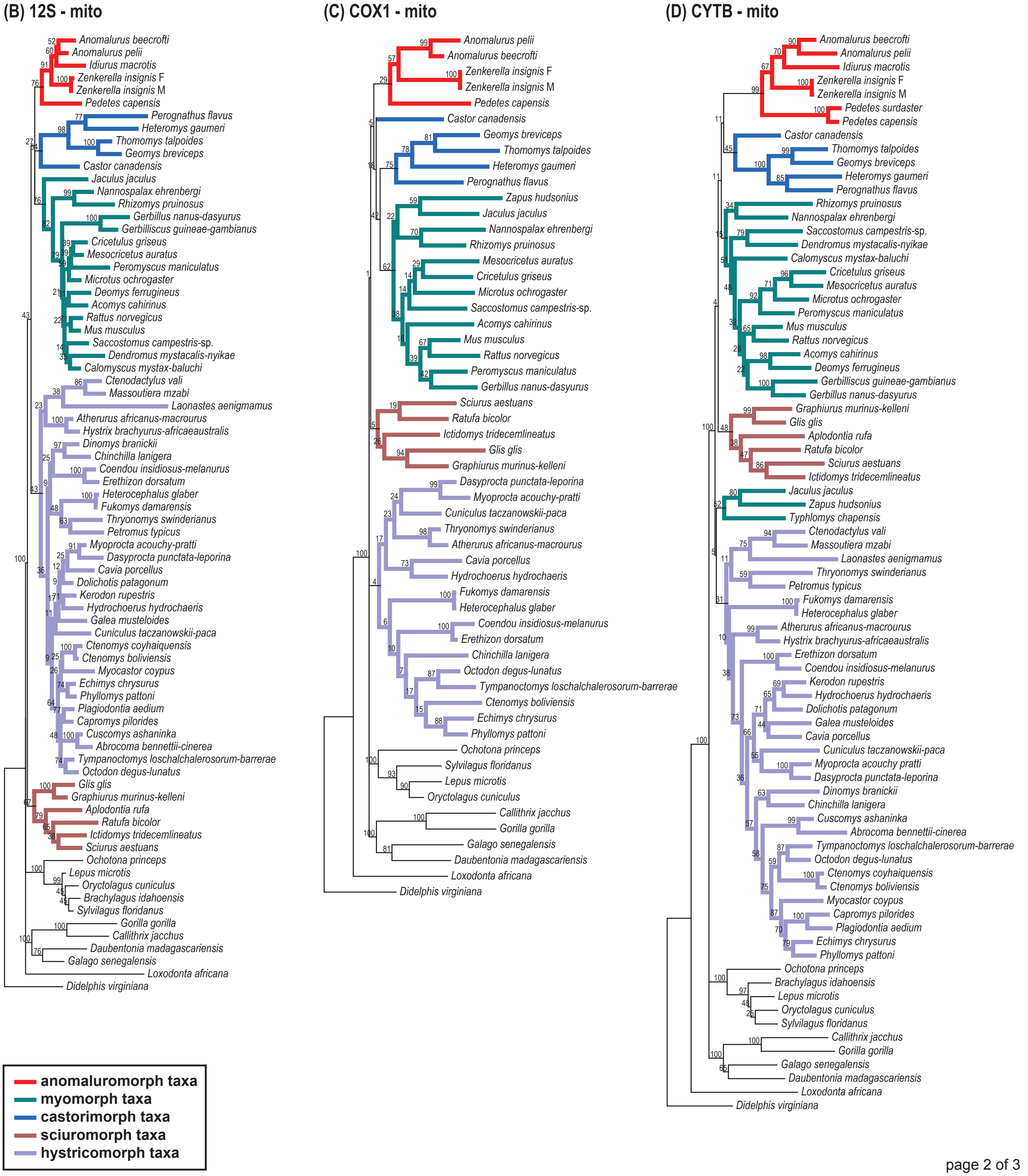

(B) 12S - mito
Anomalurus beecrofti
Anomalurus pelii
Idiurus macrotis
Zenkerella insignis F
Zenkerella insignis M
Pedetes capensis
Perognathus flavus
Heteromys gaumeri
Thomomys talpoides
Geomys breviceps
Castor canadensis
Jaculus jaculus
Nannospalax ehrenbergi
Rhizomys pruinosus
Gerbillus nanus-dasyurus
Gerbilliscus guineae-gambianus
Cricetulus griseus
Mesocricetus auratus
Peromyscus maniculatus
Microtus ochrogaster
Deomys ferrugineus
Acomys cahirinus
Rattus norvegicus
Mus musculus
Saccostomus campestris-sp.
Dendromus mystacalis-nyikae
Calomyscus mystax-baluchi
Ctenodactylus vali
Massoutiera mzabi
Laonastes aenigmamus
Atherurus africanus-macrourus
Hystrix brachyurus-africaeaustralis
Dinomys branickii
Chinchilla lanigera
Coendou insidiosus-melanurus
Erethizon dorsatum
Heterocephalus glaber
Fukomys damarensis
Thryonomys swinderianus
Petromus typicus
Myoprocta acouchy-pratti
Dasyprocta punctata-leporina
Cavia porcellus
Dolichotis patagonum
Kerodon rupestris
Hydrochoerus hydrochaeris
Galea musteloides
Cuniculus taczanowskii-paca
Ctenomys coyhaiquensis
Ctenomys boliviensis
Myocastor coypus
Echimys chrysurus
Phyllomys pattoni
Plagiodontia aedium
Capromys pilorides
Cuscomys ashaninka
Abrocoma bennettii-cinerea
Tympanoctomys loschalchalerosorum-barrerae
Octodon degus-lunatus
Glis glis
Graphiurus murinus-kelleni
Aplodontia rufa
Ratufa bicolor
Ictidomys tridecemlineatus
Sciurus aestuans
Ochotona princeps
Lepus microtis
Oryctolagus cuniculus
Brachylagus idahoensis
Sylvilagus floridanus
Gorilla gorilla
Callithrix jacchus
Daubentonia madagascariensis
Galago senegalensis
Loxodonta africana
Didelphis virginiana
(C) COX1 - mito
Anomalurus pelii
Anomalurus beecrofti
Zenkerella insignis F
Zenkerella insignis M
Pedetes capensis
Castor canadensis
Geomys breviceps
Thomomys talpoides
Heteromys gaumeri
Perognathus flavus
Zapus hudsonius
Jaculus jaculus
Nannospalax ehrenbergi
Rhizomys pruinosus
Mesocricetus auratus
Cricetulus griseus
Microtus ochrogaster
Saccostomus campestris-sp.
Acomys cahirinus
Mus musculus
Rattus norvegicus
Peromyscus maniculatus
Gerbillus nanus-dasyurus
Sciurus aestuans
Ratufa bicolor
Ictidomys tridecemlineatus
Glis glis
Graphiurus murinus-kelleni
Dasyprocta punctata-leporina
Myoprocta acouchy-pratti
Cuniculus taczanowskii-paca
Thryonomys swinderianus
Atherurus africanus-macrourus
Cavia porcellus
Hydrochoerus hydrochaeris
Fukomys damarensis
Heterocephalus glaber
Coendou insidiosus-melanurus
Erethizon dorsatum
Chinchilla lanigera
Octodon degus-lunatus
Tympanoctomys loschalchalerosorum-barrerae
Ctenomys boliviensis
Echimys chrysurus
Phyllomys pattoni
Ochotona princeps
Sylvilagus floridanus
Lepus microtis
Oryctolagus cuniculus
Callithrix jacchus
Gorilla gorilla
Galago senegalensis
Daubentonia madagascariensis
Loxodonta africana
Didelphis virginiana
(D) CYTB - mito
Anomalurus beecrofti
Anomalurus pelii
Idiurus macrotis
Zenkerella insignis F
Zenkerella insignis M
Pedetes surdaster
Pedetes capensis
Castor canadensis
Thomomys talpoides
Geomys breviceps
Heteromys gaumeri
Perognathus flavus
Rhizomys pruinosus
Nannospalax ehrenbergi
Saccostomus campestris-sp.
Dendromus mystacalis-nyikae
Calomyscus mystax-baluchi
Cricetulus griseus
Mesocricetus auratus
Microtus ochrogaster
Peromyscus maniculatus
Mus musculus
Rattus norvegicus
Acomys cahirinus
Deomys ferrugineus
Gerbilliscus guineae-gambianus
Gerbillus nanus-dasyurus
Graphiurus murinus-kelleni
Glis glis
Aplodontia rufa
Ratufa bicolor
Sciurus aestuans
Ictidomys tridecemlineatus
Jaculus jaculus
Zapus hudsonius
Typhlomys chapensis
Ctenodactylus vali
Massoutiera mzabi
Laonastes aenigmamus
Thryonomys swinderianus
Petromus typicus
Fukomys damarensis
Heterocephalus glaber
Atherurus africanus-macrourus
Hystrix brachyurus-africaeaustralis
Erethizon dorsatum
Coendou insidiosus-melanurus
Kerodon rupestris
Hydrochoerus hydrochaeris
Dolichotis patagonum
Galea musteloides
Cavia porcellus
Cuniculus taczanowskii-paca
Myoprocta acouchy pratti
Dasyprocta punctata-leporina
Dinomys branickii
Chinchilla lanigera
Cuscomys ashaninka
Abrocoma bennettii-cinerea
Tympanoctomys loschalchalerosorum-barrerae
Octodon degus-lunatus
Ctenomys coyhaiquensis
Ctenomys boliviensis
Myocastor coypus
Capromys pilorides
Plagiodontia aedium
Echimys chrysurus
Phyllomys pattoni
Ochotona princeps
Brachylagus idahoensis
Lepus microtis
Oryctolagus cuniculus
Sylvilagus floridanus
Callithrix jacchus
Gorilla gorilla
Galago senegalensis
Daubentonia madagascariensis
Loxodonta africana
Didelphis virginiana
anomaluromorph taxa
myomorph taxa
castorimorph taxa
sciuromorph taxa
hystricomorph taxa

(E) IRBP - nuc

(F) VWF - nuc

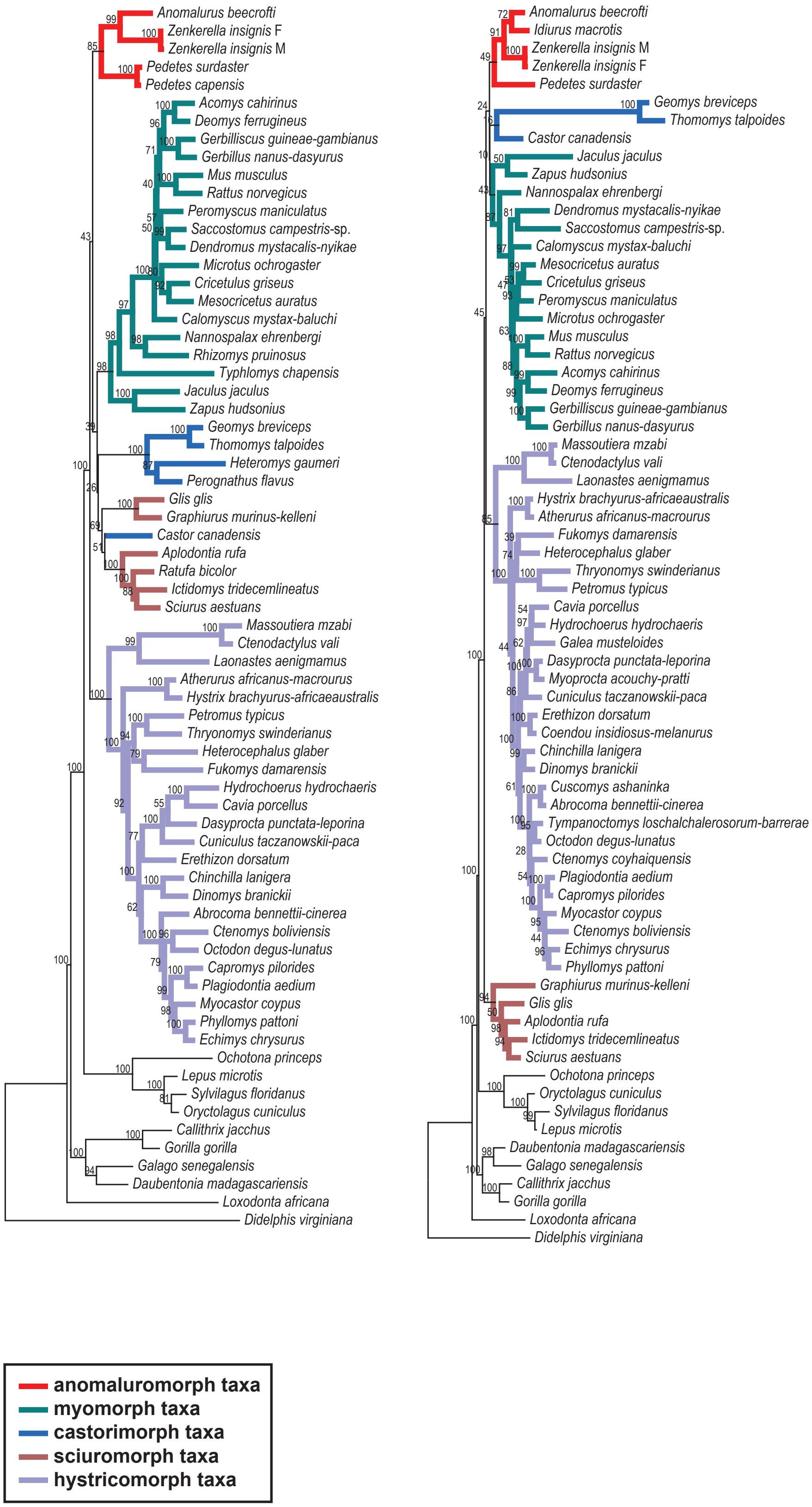

Supplement: Data S4 [file peerj-04-2320-s004.zip › HERITAGE_ET_AL_PEERJ_SUPPLEMENTAL_DATA_S4 (7-27)/Data_S4.pdf]
