# Supplementary material for: Ancient phylogenetic divergence of the enigmatic African rodent Zenkerella and the origin of anomalurid gliding
Source: PeerJ. 2016 Aug 16;4:e2320. doi: 10.7717/peerj.2320 (PMC4991859; doi:10.7717/peerj.2320)
Supplement: Data S7 [file peerj-04-2320-s007.zip › Data_S7.pdf]

Ancient phylogenetic divergence of the enigmatic African rodent *Zenkerella* and the origin of anomalurid gliding

Heritage S, Fernández D, Sallam HM, Cronin DT, Esara Echube JM, Seiffert ER (2016)

Data S7. Comparison of trees from morphology-based analyses.

We note that the characters in these morphological matrices are largely (or entirely) limited to features preserved in fossil taxa and are not explicitly formulated to inform the relationships of living anomalures. The largest difference between our combined evidence time-tree (B) and previous morphology-only trees (C and D) is the position of *Zenkerella-Prozenkerella*. Otherwise, the three trees are largely congruent. Our new Bayesian analysis (E) of the morphology-only matrix does not enforce an "extants-scaffold" yet recovers a basal position for *Zenkerella* among living taxa as in our molecular tree. However, in the absence of taxon age constraints (fossils bound to age-ranges, extants bound to zero) the fossil taxa in this result are arranged somewhat differently. Living = red, Fossil = black.

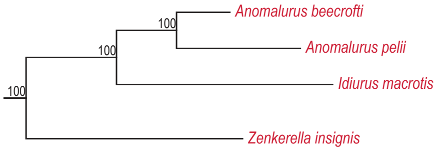

(A) Resultant subtree from our Bayesian phylogenetic analysis of the concatenated molecular alignment (i.e. subtree from our Figure 2). This topology was used as a scaffold in our the time-tree analysis. Node values are posterior probabilities.

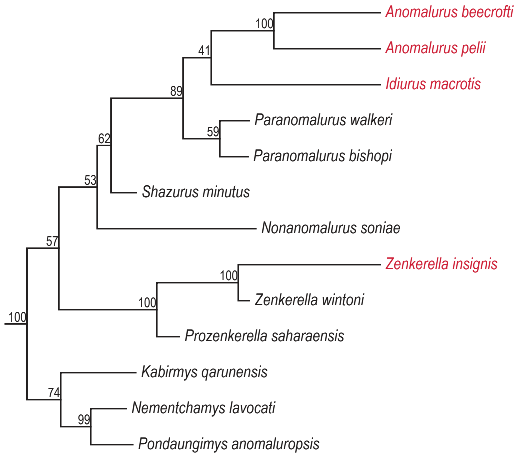

(B) Resultant subtree from our Bayesian time-tree analysis of the combined molecular and morphological dataset (i.e. subtree from our Figure 3). The molecular topology of living anomaluromorphs was used as a scaffold (soft constraint) allowing fossil taxa to be positioned anywhere on the tree. Node values are posterior probabilities.

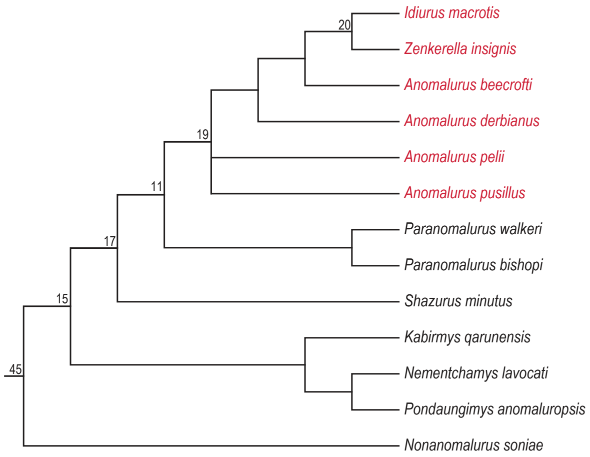

(C) Resultant subtree from the Marivaux et al. (2015) parsimony analysis of 150 (primarily dental) morphological characters. Node values are bootstrap proportions. Nodes without values are unexplained, but were presumably dropped due to a cut-off value (i.e. lower than the lowest value on this tree). [topology is from their Figure 5]

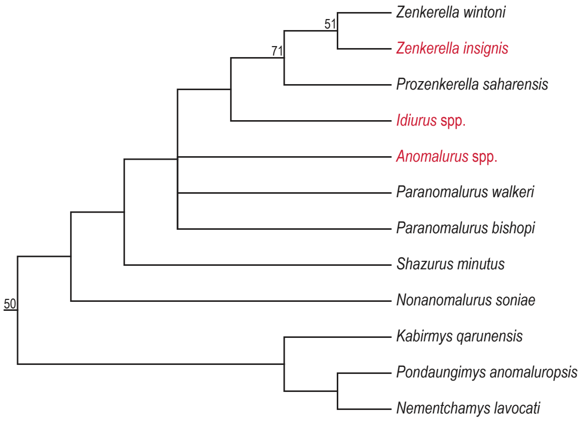

(D) Resultant subtree from the Coster et al. (2015) parsimony analysis of 108 (primarily dental) morphological characters. Node values are bootstrap proportions. Nodes without values are <50%. [topology is from their Figure 9]

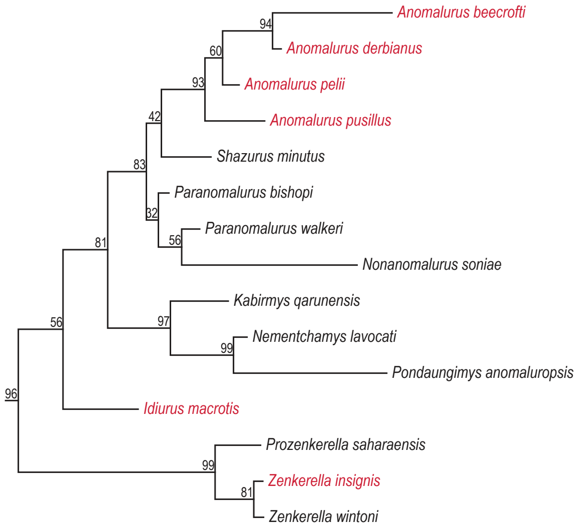

(E) Resultant subtree from our new Bayesian phylogenetic analysis of morphology-only data. The character-taxon matrix is from Marivaux et al. (2015) [150 primarily dental characters] with our addition of *Prozenkerella saharaensis* and *Zenkerella wintoni* character scores. Settings of the analysis are in a provided MrBayes commands file. No scaffold was applied. Convergence diagnostics were: Max ASDSF ~ 0.014, Max |1-PSRF| ~ 0.004, Min ESS > 6300. Node values are posterior probabilities.
